# Myocardial protection of S-nitroso-L-cysteine in diabetic cardiomyopathy mice

**DOI:** 10.3389/fendo.2022.1011383

**Published:** 2022-10-12

**Authors:** Lulu Peng, Mengying Zhu, Shengqi Huo, Wei Shi, Tao Jiang, Dewei Peng, Moran Wang, Yue Jiang, Junyi Guo, Lintong Men, Bingyu Huang, Qian Wang, Jiagao Lv, Li Lin, Sheng Li

**Affiliations:** ^1^ Division of Cardiology, Department of Internal Medicine, Tongji Hospital, Tongji Medical College, Huazhong University of Science and Technology, Wuhan, China; ^2^ Department of Geriatrics, Tongji Hospital, Tongji Medical College, Huazhong University of Science and Technology, Wuhan, China

**Keywords:** diabetic cardiomyopathy, glucose uptake, insulin signaling pathway, S-nitrosothiols, S-nitrosylation

## Abstract

Diabetic cardiomyopathy (DCM) is a severe complication of diabetes mellitus that is characterized by aberrant myocardial structure and function and is the primary cause of heart failure and death in diabetic patients. Endothelial dysfunction plays an essential role in diabetes and is associated with an increased risk of cardiovascular events, but its role in DCM is unclear. Previously, we showed that S-nitroso-L-cysteine(CSNO), an endogenous S-nitrosothiol derived from eNOS, inhibited the activity of protein tyrosine phosphatase 1B (PTP1B), a critical negative modulator of insulin signaling. In this study, we reported that CSNO treatment induced cellular insulin-dependent and insulin-independent glucose uptake. In addition, CSNO activated insulin signaling pathway and promoted GLUT4 membrane translocation. CSNO protected cardiomyocytes against high glucose-induced injury by ameliorating excessive autophagy activation, mitochondrial impairment and oxidative stress. Furthermore, nebulized CSNO improved cardiac function and myocardial fibrosis in diabetic mice. These results suggested a potential site for endothelial modulation of insulin sensitivity and energy metabolism in the development of DCM. Data from these studies will not only help us understand the mechanisms of DCM, but also provide new therapeutic options for treatment.

## Introduction

Type 2 diabetes mellitus (T2DM) is one of the most prevalent long-range metabolic disorders worldwide. It is currently spreading in an astonishing speed. From the International Diabetes Federation (IDF), it is estimated that the incidence of diabetes will reach 10.5% by 2021, up from 4.6% in 2000. By 2030, 578 million people are expected to be affected by diabetes, and 700 million people are expected by 2045 (1). Diabetic cardiomyopathy is a significant cause of morbidity and mortality in diabetic patients (2). The diagnosis of DCM is usually based on the presence of cardiac dysfunction but no hypertension or coronary artery disease in diabetic patients (3). Although many attempts have been made to research effective treatments for the long-term management of diabetic cardiomyopathy, the overall clinical results are still disappointing (4). To date, there remains an urgent need for effective treatment for diabetic cardiomyopathy.

Generally, three key pathophysiological factors (e.g., insulin resistance, endothelial dysfunction and metabolic disturbances in the myocardium) are believed to be involved in T2DM. Insulin signal transduction is a fundamental regulatory mode to ameliorate IR and myocardial energy metabolism. Through the regulation of this signaling pathway, a variety of physiological and pathological mechanisms can be improved, making it a very attractive treatment for T2DM complicated by heart failure. The insulin signal is turned on insulin binding to the insulin receptor to activate the MAPK and AKT pathways. On the other hand, insulin signaling is turned off by protein tyrosine phosphatases (PTPs), mainly PTP1B, dephosphorylation of insulin receptors and their substrates to downregulate signal transduction (5). PTP1B, an extensively expressed nonreceptor classical PTP, belongs to the class 1 PTPs (6). As required for PTP1B catalysis, the N-terminal domain contains nucleophilic residues Cys215 and Arg221. It regulates the activity of PTP1B through some posttranslational modifications (PTMs), such as phosphorylation, ER surface binding, oxidization, O-GlcNAcylation (7), sumoylation, S-nitrosylation (8) and sulfhydrylation, to perform the corresponding biological functions. Numerous PTP1B inhibitors have been reported, but few of them have been used in the clinic due to difficulties in discovering their potency *in vivo* (9). Therefore, finding a new inhibitor of PTP1B is extremely important.

Endothelial dysfunction (ED) is the mutual pathological mechanism of diabetes and cardiovascular diseases, which directly impacts the overall prognosis and survival (10). Under normal circumstances, endothelial cells maintain vascular homeostasis to maintain healthy vascular function. The endothelium mainly fights against diseases by regulating vascular tone, maintaining vascular wall permeability and antagonizing vascular inflammation (11). The endothelium lines the inner surface of vascular systems with squamous epithelial cells. A variety of mechanisms are involved in endothelial functions, including the regulation of vasodilation and contraction, thrombosis, immunity, and inflammation. Endothelial dysfunction eventually leads to vascular remodeling, myocardial remodeling, and cardiac dysfunction (12). Endothelial dysfunction is often an early pathophysiological feature of most cardiovascular diseases (CVDs) and an independent predictor of future cardiovascular prognosis (13). Clinically, the leading cause of the high morbidity of heart failure and poor prognosis in diabetic patients may be endothelial dysfunction (14). Nitric oxide (NO) plays a major role in endothelial function and is predominantly synthesized by endothelial NO synthase (eNOS). NO signaling regulates vasodilation and myocardial contraction to play a certain role in myocardial protection (15). In addition to canonical NO signaling, a recent meta-analysis of proteomic data suggested that cysteine S-nitrosylation was also a regulator of myocardial cell metabolism (16). S-nitroso-L-cysteine (CSNO) is the simplest endogenous S-nitrosothiol and is derived from eNOS. It exerts biological activity through the S-nitrosylation reaction, independent of NO. Previously, we reported that CSNO nitrosylated PTP1B to interfere with its binding to the substrate and inhibited its enzyme activity (17). In this study, we investigated the protective effect of SNO on the cardiac structure and function of DCM and reported the effect of SNO on cellular glucose uptake, the insulin signaling pathway and the potential mechanism of improving high glucose-induced injury to cardiomyocytes.

## Materials and methods

### Ethics statement

A committee of Tongji Medical College, Huazhong University of Science and Technology approved the use of wild-type C57BL/6J mice (male, 4 weeks and 15 g). All procedures were executed according to the National Institutes of Health Guide for the Care and Use of Laboratory Animals.

### Animals and treatment

C57BL/6J mice (Shulaibao Biotech) were permitted to adapt to feed in the experimental environment for 2 weeks. The DCM model was developed by following a high-fat diet for 8 weeks and injecting STZ (150 mg/kg, Sigma−Aldrich, USA) intraperitoneally for 5 days. The control group was given the same amount of citrate buffer (vehicle) intraperitoneally and fed a normal diet. After 1 week, blood glucose measurements were monitored. Three consecutive random measurements of blood glucose above 16.7 mM were considered successful. The early treatment group (ET group) was administered nebulized CSNO (88 ppm for 20 minutes per day) when fed a high-fat diet, while the late treatment group (LT group) was administerednebulized CSNO when the model was built.

### Doppler echocardiography

Each group of mice was examined using echocardiography at week 2, and 16. 3% isoflurane mixed in 1 L/min 100% O_2_ was used to induce anesthesia in mice, and 2% isoflurane mixed in 1 L/min 100% O_2_ was used to maintain anesthesia. We averaged the cardiac parameters from at least three separate cardiac cycles. For echocardiographic operators, the grouping of mice was blinded.

### Histological staining

Paraffin sections were prepared by agar preembedding myocardial tissue. The blocks were dyed with hematoxylin-eosin (HE), Sirius Red and Masson’s trichrome. In addition, the area of myocardial cells was quantified by staining with wheat germ agglutinin (WGA). This staining was performed using standard protocols.

### Cell culture

H9c2 myoblasts (ATCC, Manassas, U.S.) were cultured in Dulbecco’s modified Eagle’s medium (KeyGEN BioTECH, China) supplemented with 10% (v/v) fetal bovine serum (FBS, Gibco, Thermo Fisher Scientific, U.S.) and 1% (v/v) penicillin/streptomycin (Sangon, China) in an incubator (5% CO_2_, humidified at 37°C). Cells were induced with high-glucose DMEM (33 mM) configured with anhydrous glucose for 48 h to simulate hyperglycemic conditions.

### Determination of cell viability

The cells were seeded in 96-well plates at 5 × 10^3^ cells per well. When the cells grew for approximately 12 h and adhered well, they were exposed to different concentrations of CSNO for 1 h. CCK8 assays were used to determine cell viability and growth. At a wavelength of 450 nm, the absorbance was detected by a spectrophotometer.

### Glucose uptake assay

After treatment, the cells were coincubated with ^3^H labeled 2- deoxyglucose (^3^H-2-DOG). After washing the cells, the ^3^H-2-DOG content in cardiomyocytes was measured by a scintillation counter, and glucose uptake was quantitatively detected.

### Western blot

The treated cells and spilt cells were collected using RIPA lysis buffer (18). Equal amounts of protein were loaded on Bis-Tris SDS−PAGE (the concentration was determined by the molecular weight of protein) for gel electrophoresis. The band was transferred to PVDF membrane and combined with antibody overnight. The antibodies we used were as follows: p-Akt (CST, #4060, 1:1000), AKT (CST, #4685, 1:1000), p-IR (CST, #3021, 1:1000),IR (CST, #23413, 1:1000), IRS (CST, #95816, 1:1000), p-IRS (Sigma−Aldrich, ZRB09432, 1:1000), GLUT4 (CST, #2213, 1:1000), N-cadherin (CST, #13116, 1:1000), LC3B (CST, #3868, 1:1000), P62 (CST, #23214, 1:1000) and GAPDH (CST, #2118L). A prestained protein ladder (Thermo Scientific) and HRP-conjugated secondary antibodies were applied as well.

### Real-time PCR

We conducted a quantitative PCR study as described in our recent publication (18). The primer sequences were as follows: Mouse ANP Forward 5’-ACCTGCTAGACCACCTGGAG-3’, Reverse 5’-CCTTGGCTGTTATCGGTACCGG-3’. Mouse BNP Forward 5’-GAGGTCACTCCTATCCTCTGG-3’, Reverse 5’-GCCATTTCCTCCGACTTTTCTC-3’. Mouse β-MHC Forward 5’-CCGAGTAGGTCAACAA-3’, Reverse 5’-CTTCACGGGCACCCTTGGA-3’. Mouse COL-1 Reverse 5’-AGGCTTCAGTGGTTTGGATG-3’, Reverse 5’-CACCAACAGCACCATCGTTA-3’. Mouse GAPDH’Forward 5’-GGTTGTCTCCTGCGACTTCA-3’, Reverse 5’-TGGTCCAGGGTTTCTTACTCC-3’. To calculate fold changes in gene expression, ΔCt test = Ct of target genes in test groups - Ct of gapdh in test group and ΔCt con = Ct of target genes in control groups - Ct of gapdh in control group were first obtained. Next, the ΔCt test was normalized by ΔCt con. ΔΔCt = ΔCt test - ΔCt con. The final step is to calculate the difference in expression levels. Change fold = 2^(-ΔΔCt).

### Plasma membrane separation experiment

A membrane protein extraction kit (Beyotime, China) was used to extract cell membrane proteins. The isolated protein was detected by western blot.

### Measurement of ATP contents

The ATP levels were measured using an enhanced ATP assay kit (Beyotime S0027). The assays were performed according to the instruction manual. A luminometer was used to measure the optical density of the mixture. Based on an ATP concentration-versus-absorptance standard curve, the ATP concentration was calculated.

### Mitochondrial membrane potential assay

After cell treatment, the culture medium was removed and discarded. The cells were washed with preheated PBS 3 times. In accordance with the instructions provided, treated cells were incubated with 200 nM JC-1 (C2006, Beyotime) for 30 minutes at 37°C. The fluorescence of red and green was detected using a fluorescence microscope. MMP fluorescence intensity was assessed using ImageJ software. MMP =JC-1 polymer fluorescence intensity/JC-1 monomer fluorescence intensity.

### Mitochondrial morphological measurement

A density of 1 × 10^5^ cells/dish seeded in confocal dishes was treated with high glucose (33 mM) for 48 h and CSNO (50 μM) for 1 h. Three rinses with PBS were followed by 30 min of incubation with 200 nM MitoTracker^®^ Red CMXRos (Thermo Fisher). Image capture of mitochondria was performed using confocal laser scanning microscopy (Eclipse-TI2, Nikon, Japan). An analysis of mitochondrial morphology was performed by using ImageJ with Mitochondrial Network Analysis (MiNA). The following three parameters were used to evaluate the morphology of the mitochondrial network: individuals of mitochondria, mitochondrial footprint and mean network size.

### Measurement of cardiomyocyte size

After being exposed to high glucose (33 mM) for 48 h and CSNO (50 μM) for 1 h, H9c2 were fixed with 4% paraformaldehyde for 15 min. Following washing with TBST 3 times, the cells were blocked with a 10% solution of normal goat serum in PBS for 1 h. Then, the slides were incubated with 50 μg/mL phalloidin (AAT BioQuest, #23122) for 1 h away from light. DAPI counterstain (Sigma−Aldrich, MO, USA) was applied to the nuclei of each cell. Images capture of cell shape was performed by laser scanning confocal microscopy (Eclipse-TI2, Nikon, Japan).

### Autophagic flux assay

Tandem mRFP-GFP-LC3 overexpression was achieved by infection of cells (a density of 1 × 10^5^ cells/dish seeded in confocal dishes) with recombinant adenoviruses (Genechem, Shanghai, China) for 10 h after attachment. Fluorescence was checked by fluorescence microscopy to determine whether the virus had been transferred into the cells. After the virus was transferred into the cells, the cells were washed with PBS and cultivated in high-glucose for 48 h and CSNO for 1 h. Confocal laser scanning microscopy (Eclipse-TI2, Nikon, Japan) was used to capture autophagy flux images.

### Statistical analysis

Data were expressed as an SEM average in triplicate. ImageJ software was used to quantitatively analyze the results of histological staining, WB and immunofluorescence. The statistical analysis and drawing were carried out using SPSS 21.0 software and GraphPad 8.0. We used one-way and two-way ANOVA to determine whether there were significant differences between the experimental groups. The threshold for statistical significance was p < 0.05.

## Results

### Effects of SNO on the metabolic parameters of diabetic mice

**Figure 1:**
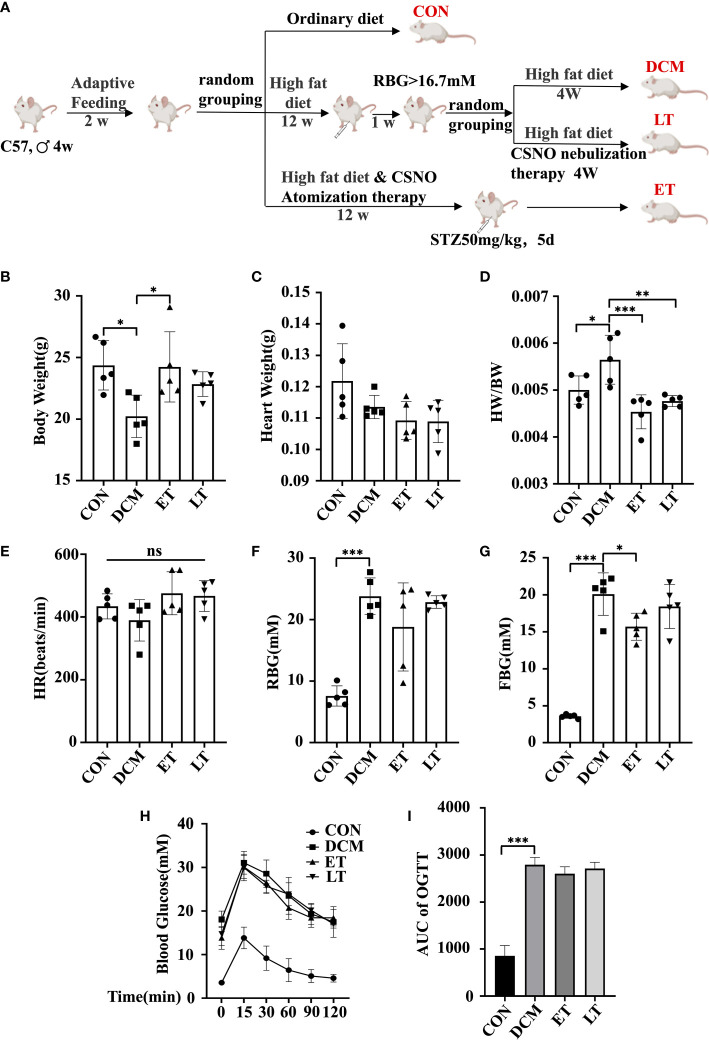
Mouse body weight, heart weight and blood glucose levels. **(A)** A flowchart showing the experimental process for modeling mice. **(B)** Body weight of mice. **(C)** Heart weight of mice. **(D)** Heart weight to body weight ratio (HW/BW). **(E)** Heart rate (HR). **(F)** Random body glucose(RBG). **(G)** Fasting blood glucose levels (FBG). **(H)** After fasting for 16 hours, mice were given glucose solution by gavage, and the corresponding blood glucose values were measured at different time points. The corresponding curve was drawn, namely, an oral glucose tolerance test (OGTT) curve. **(I)** The area under the curve (AUCglucose) was quantified. * represents p < 0.05; ** represents p < 0.01; *** represents p < 0.001. The “ns” symbol stands for “no significance”.

To study the impact of exogenous SNO *in vivo*, we nebulized CSNO. Experimental flow chart for the experiments is shown in **Figure 1A**. There was no obvious difference in body or heart weight among the groups (**Figures 1B, C**). However, the DCM group showed an increasd heart weight/body weight (HW/BW) ratio (**Figure 1D**). These data indicated that exogenous SNO ameliorated myocardial hypertrophy in diabetic mice. There were no significant differences between the 4 groups in heart rate (HR) (**Figure 1E**). Compared with the control group, the DCM group showed higher fasting blood glucose and random blood glucose. CSNO treatment decreased fasting blood glucose but had no obvious effect on random blood glucose (**Figures 1F, G**). As shown in **Figure 1H**, OGTT testing showed blood glucose levels of DCM mice spiked after glucose administration (15 min) and remained high after 120 min. The AUC_0−120 minGlu_ levels were significantly increased in the T2DM group after the dietary intervention start, while the ET group and LT group were lower (**Figure 1I**). These data indicate that exogenous SNO improved the glucose homeostasis of diabetic mice.

### SNO ameliorated cardiac dysfunction in diabetic mice

**Figure 2:**
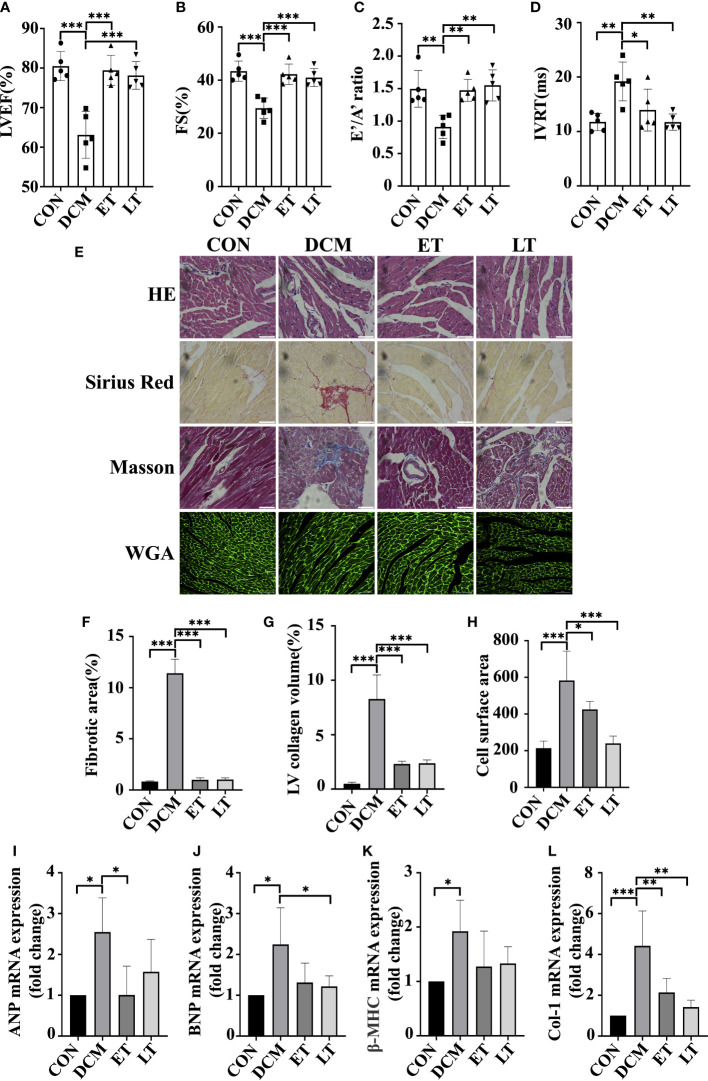
The effect of SNO on cardiac function in diabetic mice. Indices of cardiac systolic function, **(A)** Left ventricular ejection fraction (EF %). **(B)** Fractional shortening (FS %). Indices of cardiac diastolic function, **(C)** Early-to-late septal annulus motion in diastole (E’/A’) and **(D)** Isovolumic relaxation time (IVRT). **(E)** HE staining, Sirius red staining, Masson trichrome staining and WGA staining of mouse hearts. Scale bar=50 mm. **(F)** The percentage of fibrotic area was quantified. **(G)** The percentage of LV collagen area was quantified. **(H)** Quantitative analysis of cardiac myocyte cross-sections. **(I-L)** Relative ANP, BNP, β-MHC and Col-1 mRNA expression normalized to GAPDH. * represents p < 0.05; ** represents p < 0.01; *** represents p < 0.001.

DCM is diagnosed by detecting both structural and functional alterations in the left ventricle (LV) (19). Using M-mode echocardiography, we assessed the protective effect of exogenous SNO on the cardiac function of DCM mice. In comparison with the control group, a significant decrease in ejection fraction (EF) and fractional shortening (FS) was observed in DCM animals, which showed poor systolic dysfunction, indicating that DCM model was successfully developed. However, CSNO treatment markedly restored the aberrant myocardial parameters (**Figures 2A, B**). In addition, CSNO treatment reduced the E’/A’ ratio and IVRT compared with DCM mice, indicating that CSNO improved diastolic function (**Figures 2C, D**). Staining with HE, Masson’s trichrome, Sirius red, and WGA was used to evaluate the changes in the myocardium in DCM mice. The results showed that DCM mice exhibited disarrayed cellular structures, ruptured myofibrils, increased fibrotic infiltration and obscured intercellular borders (**Figures 2E–G**). WGA staining showed increased cardiomyocyte size, and CSNO treatment reduced cardiomyocyte size (**Figure 2H**). All these anomaly variations were mitigated by CSNO treatment in the ET group and LT group. We also determined the mRNA expression levels of ANP, BNP, β-MHC, and col-1 by quantitative real-time PCR. Consistent with the results described previously, there was an ameliorative effect of CSNO on myocardial fibrosis and myocyte hypertrophy (**Figures 2I–L**).

### SNO increased cellular glucose uptake

**Figure 3:**
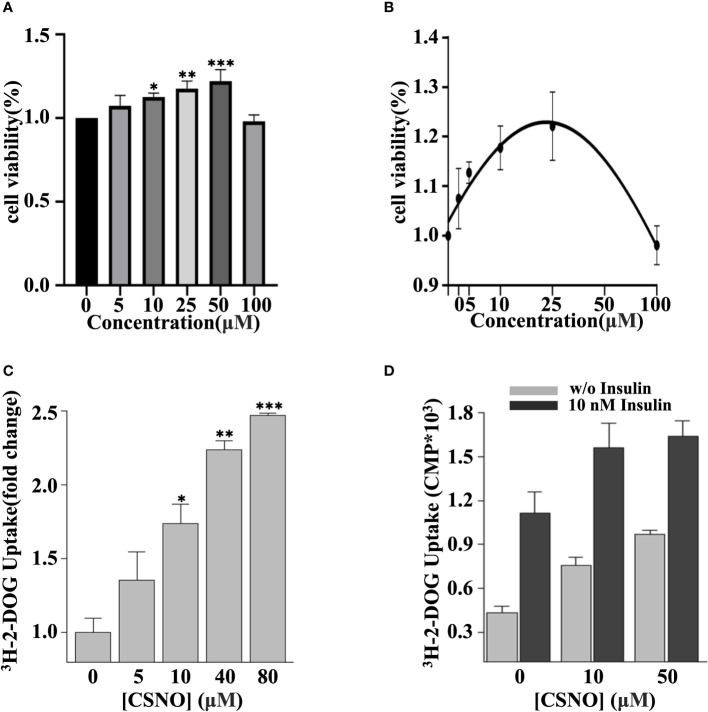
CSNO promoted cellular glucose uptake. **(A)** H9c2 cells were incubated with CSNO at different concentrations for 1 h. Cell viability was examined using a CCK8 kit. **(B)** The corresponding curve diagram of cell viability. **(C)** CSNO promoted cellular glucose uptake in a concentration-dependent manner. **(D)** CSNO increased insulin-dependent and noninsulin-dependent glucose uptake. * represents p < 0.05; ** represents p < 0.01; *** represents p < 0.001.

To investigate the effects of exogenous SNO on cardiomyocyte viability, H9c2 cells were exposed to CSNO at different concentrations for 1 h. As revealed in **Figures 3A, B**, 100 μM CSNO treatment or a lower concentration had no obvious effect on cytotoxicity. CSNO treatment significantly enhanced cell viability at low concentrations, and this effect was concentration dependent. A disturbance in glucose metabolism is the key point of the development of DCM. Although diabetic patients are in a hyperglycemic state, due to insulin resistance, the intake of glucose is insufficient. The cells are actually in a "hungry" state. Therefore, it is essential to restore insulin sensitivity and increase glucose intake to regulate glucose homeostasis. To test the effect of SNO on glucose uptake, cells were pretreated with different concentrations of CSNO with or without insulin. The results showed that CSNO alone increased glucose uptake in a dose-dependent manner (**Figure 3C**). In short, CSNO had an insulin-like effect. CSNO also increased glucose uptake in the presence of insulin, which indicated that CSNO increased insulin sensitivity (**Figure 3D**).

### SNO enhanced GLUT4 translocation in cardiomyocytes

**Figure 4:**
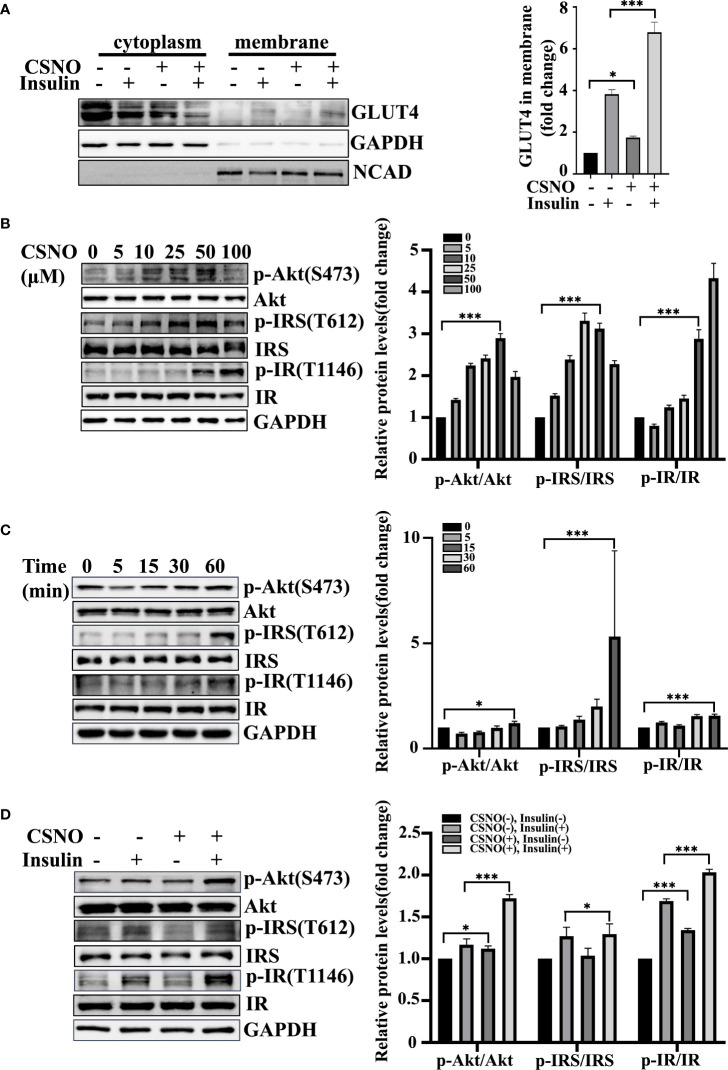
CSNO promoted GLUT4 membrane translocation and the insulin signaling pathway. **(A)** After the cytoplasm and cell membrane were separated, the expression of GLUT4 on membrane protein was detected. It was normalized to N-cadherin. **(B)** CSNO concentration-dependently activated the insulin signaling pathway in H9c2 cells. The expression level and phosphorylation level of Akt, IRS and IR were analyzed by Western blot. The protein band diagram and quantitative statistical diagram are shown. **(C)** CSNO time-dependently activated the insulin signaling pathway in H9c2 cells. The expression level and phosphorylation level of Akt, IRS and IR were analyzed by Western blot. The protein band diagram and quantitative statistical diagram are shown. **(D)** CSNO promoted activation of the insulin signaling pathway and further increased the effect of insulin on the insulin signaling pathway. The expression level and phosphorylation level of Akt, IRS and IR were analyzed by Western blot. The protein band diagram and quantitative statistical diagram are shown. * represents p < 0.05; *** represents p < 0.001.

Life relies primarily on glucose as an energy source. GLUT4 is the dominant glucose transporter in the human heart (20). GLUT4 primarily resides in intracellular membrane compartments under basic conditions. A 10-to-20 fold increase in glucose transport into cardiomyocytes occurs when GLUT4 is translocated to the cell surface (21). The stimulation of myocardial glucose uptake induced by CSNO may be dependent on glucose transporter trafficking. In **Figure 4A**, the plasma membranes were separated and GLUT4 was measured. We detected that CSNO treatment not only promoted GLUT4 membrane translocation but also enhanced insulin-stimulated GLUT4 translocation.

### SNO regulated the insulin signaling pathway in cardiomyocytes

Energy metabolism disorder and severe insulin resistance (IR) are the main pathological mechanisms of T2DM complicated with heart failure. Because of the great importance of the insulin signal transduction pathway in insulin resistance, we further elucidated the effect of CSNO on the insulin signaling pathway. According to the results, CSNO treatment upregulated the expression of phospho-IR, phospho-IRS1, and phospho-Akt in a concentration/time-dependent manner (**Figures 4B, C**). In **Figure 4D**, the results showed that CSNO not only activated insulin signaling without insulin, but also further enhanced the insulin-stimulated activation of insulin signaling pathway. In conclusion, CSNO activated the insulin signaling transduction.

### SNO ameliorated high glucose-induced insulin resistance in cardiomyocytes

**Figure 5:**
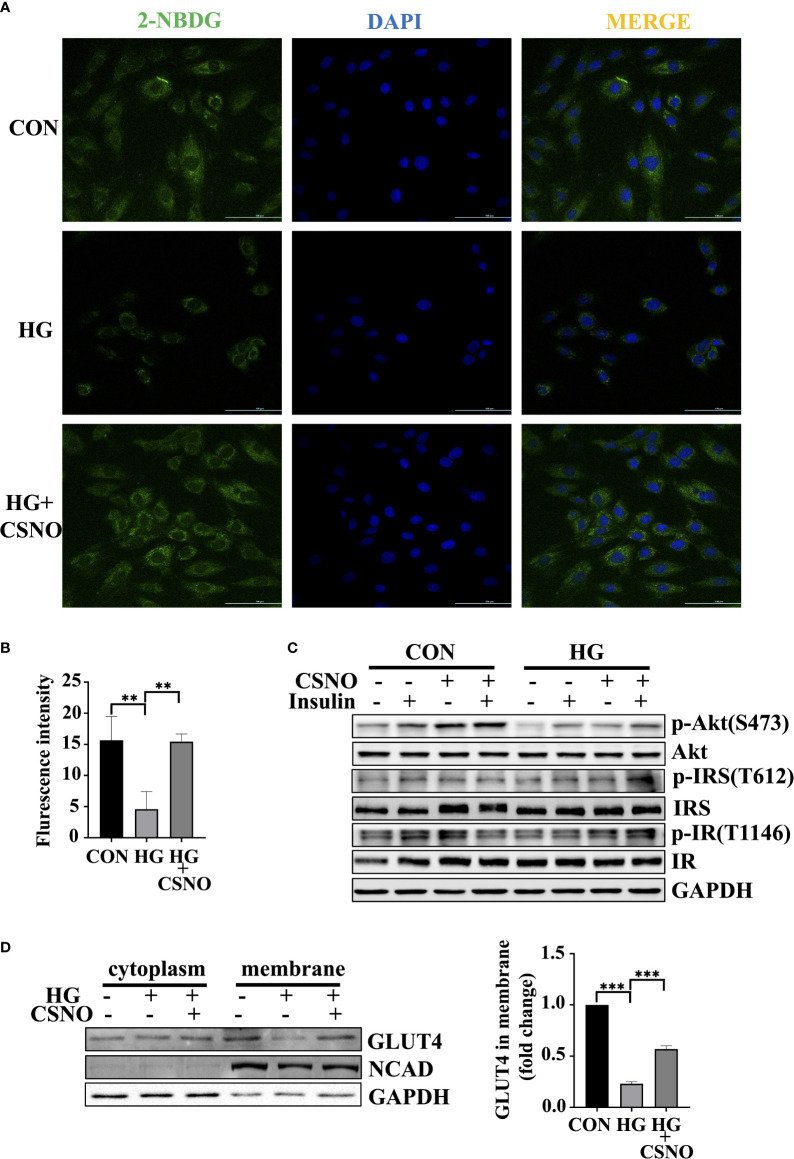
CSNO alleviated insulin resistance. **(A)** Glucose uptake was detected by fluorescence analysis of 2-NBDG uptake by cells. Scale bar=100 μm. **(B)** The statistical graph of fluorescence intensity which was analyzed with imageJ software. Scale bar=100 μm. **(C)** The expression level and phosphorylation level of Akt, IRS and IR were analyzed by Western blot. **(D)** After the cytoplasm and cell membrane were separated, the expression of GLUT4 on membrane protein was detected. It was normalized to N-cadherin. ** represents p < 0.01; ***represents p < 0.001.

High glucose for 48 h is used to model insulin resistance. Glucose uptake is defective under insulin resistance conditions. Upon high glucose stimulation, a significant reduction in glucose uptake was observed. CSNO treatment increased glucose uptake (**Figures 5A, B**). In addition, we detected that CSNO significantly activated the insulin signaling pathway under insulin resistance (**Figure 5C**). In comparison with the control group, the membrane translocation of GLUT4 stimulated by insulin decreased in states of insulin resistance. However, CSNO facilitated GLUT4 translocation (**Figure 5D**).

### SNO reversed high glucose-induced excessive autophagy in cardiomyocytes

**Figure 6:**
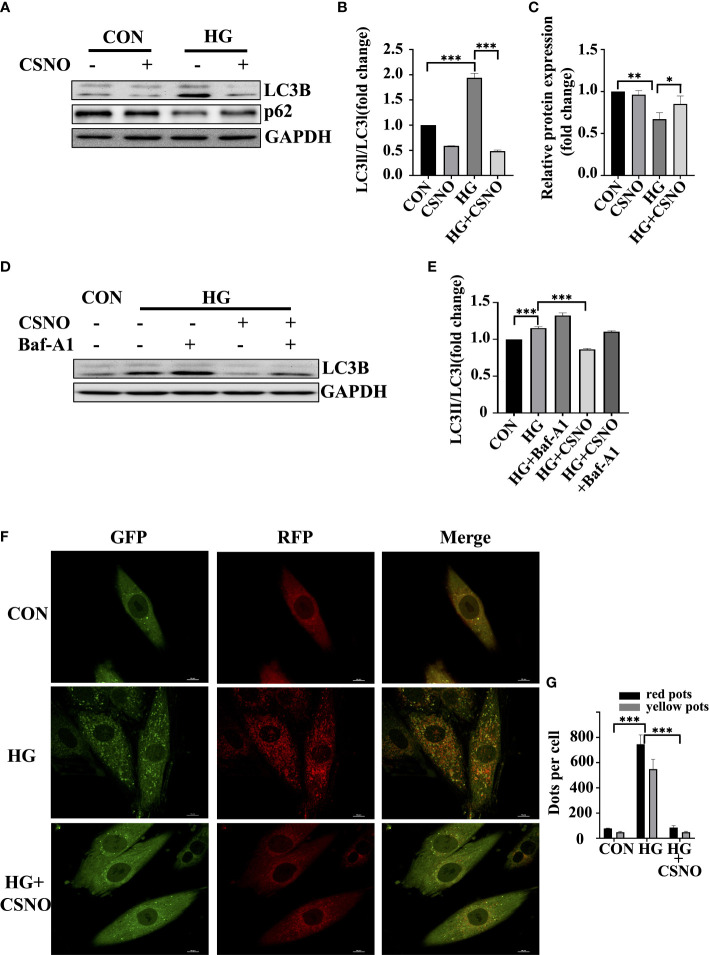
CSNO inhibited high glucose-induced excessive autophagy. **(A-C)** The expression levels of P62, LC3 II/I and GAPDH were analyzed by Western blot. The protein band diagram and quantitative statistical diagram are shown. **(D, E)** After adding the autophagy inhibitor Baf-A1,the expression of LC3 II/I and GAPDH was analyzed by Western blot. The protein band diagram and quantitative statistical diagram are shown. **(F)** An adenovirus expressing GFP-RFP-LC3 protein was used to detect autophagic flux. Scale bar=10 μm. **(G)** To quantify yellow and red LC3 dots per cell in each condition, Image-Pro Plus was used. * represents p < 0.05; ** represents p < 0.01; *** represents p < 0.001.

Autophagy is a fundamental catabolic mode that plays a very important role in maintaining cellular metabolic homeostasis by degrading and recovering some harmful components. However, the excessive activation of autophagy leads to cell death as well (22). Light chain protein 3 (LC3) is a specific marker of autophagy initiation. The P62/SQSTM1 (sequetosome1) protein is a ubiquitin-binding protein involved in the degradation of autophagy-lysosome. The accumulation of P62 indicates impaired autophagy (23). In **Figures 6A–C**, high glucose induced autophagy, evidenced by an increase in LC3-I conversion to LC3-II and a decrease in P62. CSNO treatment reversed this condition. To further clarify the effect of CSNO on autophagy, we used the autophagy inhibitor, bafilomycin A1 (Baf-A1). Baf-A1 is a lysosomal inhibitor that obstructs autophagy-lysosomal fusion and blocks autophagic flux. Baf-A1 alone caused the accumulation of LC3-II. Treatment with CSNO reduced the levels of LC3II, indicating that CSNO possibly played a role by inhibiting the formation of autophagosomes rather than blocking the degradation of autophagosomes (**Figures 6D, E**). We further used tandem fluorescent mRFP*-*GFP*-*LC3 adenovirus transfection to independently investigate the accumulation of typical autophagosomes and autolysosomes. When autophagosomes merge with lysosomes, GFP is quenched because of the acidic environment. Thus, green puncta represent autophagosomes, red puncta represent autolysosomes, yellow puncta (overlay of green and red) represent autophagosomes. Consistent with the previous results, high glucose stimulation significantly induced red puncta and yellow LC3-II puncta formation, supporting increased autophagic flux. CSNO restored autophagic flux by blocking autophagosome formation (**Figures 6F, G**).

### SNO improved high glucose-induced mitochondrial function in cardiomyocytes

**Figure 7:**
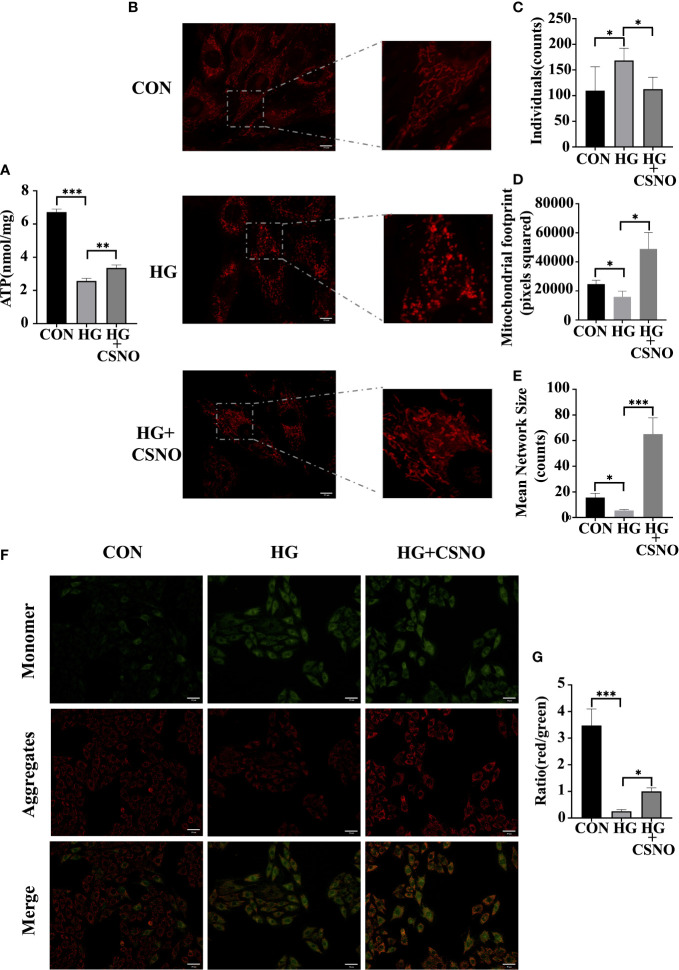
CSNO ameliorated high glucose-induced mitochondrial dysfunction. **(A)** ATP assay kits were used to determine cellular ATP levels. **(B)** Analysis of mitochondria stained with MitoTracker using confocal microscopy. Scale bar =10 μm. **(C-E)** MiNa in ImageJ was used to analyze the structural characteristics of the mitochondrial network, including individuals, mitochondrial footprint and mean network size. **(F)** Mitochondrial membrane potential (MMP) was determined by staining with JC1. JC-1 aggregates emit red fluorescence in healthy mitochondria with polarized inner mitochondria. As MMP dissipates, cytosolic monomers of JC-1 emit green fluorescence. Scale bar=50 μm. **(G)** A fluorescence ratio of red to green was used to calculate the MMP of cardiomyocytes for each group. * represents p < 0.05; ** represents p < 0.01; *** represents p < 0.001.

The major hallmark of diabetes is hyperglycemia, which perturbs the energy metabolic milieu. It impairs mitochondrial function and eventually leads to cardiac dysfunction. Compared with the control group, after high glucose stimulation for 48 h, the production of ATP decreased (**Figure 7A**), which may be related to mitochondrial destruction, and CSNO improved this effect. To observe the changes in mitochondria, MitoTracker Red was used. Under normal conditions, healthy cells showed a tubular mitochondrial network. When cells were stimulated with high glucose, mitochondrial reticulation was destroyed. The mitochondria became spherical and more fragmented, indicating an increase in mitochondrial fission. Compared with the control group, we observed that the volume of mitochondria was smaller. CSNO improved the mitochondrial morphological changes and significantly increased the volume, indicating that CSNO promoted mitochondrial fission in high glucose-treated cardiomyocytes (**Figures 7B–E**).

Additionally, maintaining mitochondrial membrane permeability and mitochondrial function is also dependent on mitochondrial membrane potential (MMP). JC-1 cationic dye was used to detect MMP. Red fluorescence represents aggregates, green fluorescence represents monomers, and the ratio of aggregates to monomers is the MMP level. MMP levels were diminished in the high glucose-treated group, whereas MMP levels were recovered with CSNO supplementation (**Figures 7F, G**).

### SNO suppressed high glucose-induced oxidative stress in cardiomyocytes

**Figure 8:**
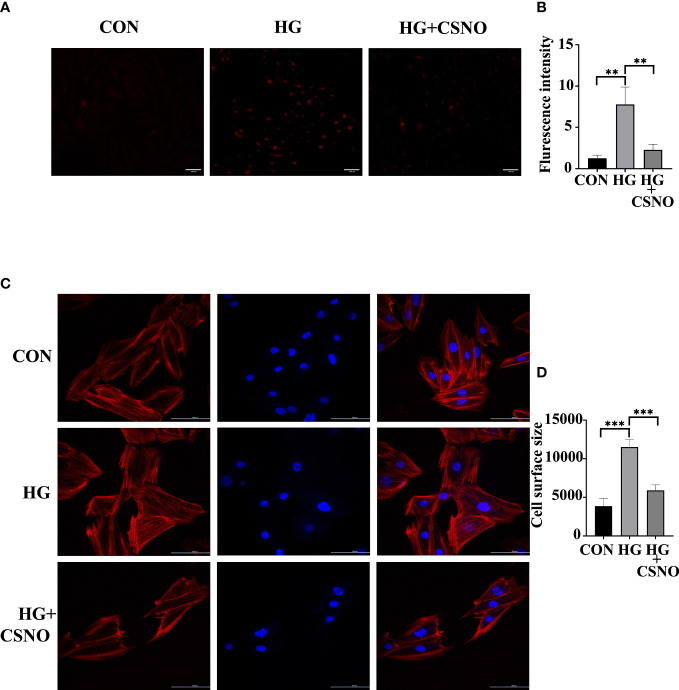
CSNO promoted high glucose-induced oxidative stress. **(A, B)** Analysis of reactive oxygen species (ROS) by dihydroethidium (DHE). Scale bar =100 μm. **(C, D)** The size of the cell was determined by detecting filaments of F-actin in the cell cytoskeleton using phalloidin. Scale bar=100 μm. ** represents p < 0.01; *** represents p < 0.001.

Oxidative stress also serves as an important pathological mechanism of DCM. Mitochondrial dysfunction can also lead to excessive production of ROS. The imbalance between ROS production and elimination is referred to as oxidative stress. To determine whether CSNO affected oxidative stress, we used DHE staining to detect intracellular ROS. As shown in **Figures 8A, B**, under the stimulation of high glucose, the intracellular fluorescence intensity obviously increased, and the CSNO treatment group decreased, indicating that CSNO alleviated the oxidative stress caused by high glucose. It has been reported that oxidative stress is essential for the development of cell hypertrophy. We stained cells with fluorescently labeled phalloidin to observe cardiomyocyte size. We found that CSNO ameliorated high glucose-induced cell hypertrophy (**Figures 8C, D**).

## Discussion

In our study, we found that an endogenous endothelial-derived SNO promoted glucose uptake in cells and increased insulin sensitivity. To the best of our knowledge, this has not been previously reported. Our results yield novel insight into how endothelial dysfunction may cause insulin resistance. Numerous studies have found that endothelial cells play an unexpected role in metabolic homeostasis, in addition to maintaining vascular homeostasis (15). In endothelial cells, GLUT1 is the primary glucose transporter. Chronic exposure to high glucose levels reduced endothelial GLUT1 expression to minimize the glucose transport rate in the heart (24). HIF1α in endothelial cells also played a role in glucose uptake, as evidenced by endothelial-specific HIF1α knockout mice exhibiting reduced CSF/blood glucose ratios (25). Insulin improved insulin sensitivity and responsiveness of nonobese people through nitric oxide-dependent vasodilation in skeletal muscle (26). According to our study, endothelial SNO activated insulin signaling pathways and facilitated GLUT4 membrane translocation to improve glucose metabolism in cardiomyocytes. It was also believed that endothelial cells were responsible for maitaining metabolic homeostasis by secreting factors such as nitric oxide (NO), insulin-stimulating factors, growth factors, and enzymes (15). Our research revealed that SNO also had the potential to maintain metabolic homeostasis, which was previously unknown.

The mechanism of DCM has been extensively studied, including abnormal energy metabolism, abnormal subcellular composition and endothelial dysfunction (27). However, there is no specific pharmaceutical treatment at present. Many studies have shown that cardiac microvascular endothelial dysfunction often appears in the first stage of DCM and runs through the whole process (28). Creating specific modifications to target certain biomolecules on microvascular endothelial cells (MVECs) and myocardial cells exposed to high glucose stimulation has shown beneficial effects in clinical trials (29). In the heart, there is a close relationship between endothelial cells and cardiac myocytes. Many cardiac activity factors derived from the endothelium can regulate the activity of cardiac myocytes, such as endothelin -1 (ET-1), neuroregulatory protein 1 (NRG-1), nitric oxide (NO) and prostaglandins, as well as the recently reported angiopoietin, neuroregulatory protein 1 (NRG-1), apelin and dickkopf-3 (30). Zhao et al. proved that ET-1 maintained normal cardiac function and myocardial survival of mice by upregulating NF-κB signaling to reduce TNF-related apoptosis (31). Apelin regulated cardiomyocytes by binding its GPCR receptor APJ to antagonize the renin-angiotensin system (32). Prostaglandin I2 improved ET1-induced myocardial hypertrophy by activating IP prostaglandin-like receptor and cyclic adenosine monophosphate-dependent signal transduction in myocardial cells (33). NO maintained normal cardiac pump function by activating the NO/cGMP/PKG pathway. NO is mainly produced by eNOS. In-depth research has found that the endothelial relaxing factor produced by eNOS is not only NO gas but also sulfhydryl nitric oxide (SNO) combined with sulfhydryl (-SH). Our results showed that SNO not only activated the insulin signaling pathway, but also protected cardiomyocytes from the damage caused by high glucose by improving abnormal energy metabolism, mitochondrial dysfunction, excessive production of reactive oxygen species and excessive autophagy. *In vivo*, nebulization of SNO in diabetic mice effectively improved cardiac dysfunction and myocardial fibrosis. It also suggested that endothelial dysfunction played an important role in DCM. However, there is still a need for further exploration of the underlying mechanism.

SNO not only has the same biological characteristics as NO gas but also regulates protein function through S-nitrosylation of thiols, which is a posttranslational modification, while NO gas has no such function. Our previous study found that SNO from eNOS inhibited PTP1B activity. PTP1B is the major negative regulator of the insulin signaling pathway. Recently, increasing attention has been focused on targeting PTP1B inhibitors for T2DM treatment. However, they have not been used in the clinic and have just reached the stage of clinical trials due to some limitations related to the tissue-specific functions of PTP1B (34). Our results shed new light on finding endogenous PTP1B inhibitors instead of chemical synthesis. Different from synthetic compounds, SNO is an endogenous molecule that already exists *in vivo*. Besides the adverse reactions caused by high concentrations, the possibility of unpredictable toxicity and side effects is small. In addition, 50–80% homology between catalytic sites of PTP1B and other phosphatases limits the use of selective PTP1B inhibitors (35). However, unlike other tyrosine phosphatases, the amino acid sequence composition and special three-dimensional structure of PTP1B in its enzyme active site, especially the basic amino acid residues, Arg45, Lys116 and Lys210, are similar to C215 in the enzyme active site in the spatial structure. Therefore, the entropy value of the nucleophilic reaction with SNO is low, which gives SNO high specificity in inhibiting PTP1B (36). To bind to the highly charged catalytic sites of PTP1B, inhibitors should be charged with anions at physiological pH, but they often show limited cell permeability and low bioavailability (37) (38);. Our previous research proved that SNO entered into cells *via* LAT1 and LAT2 to exert its corresponding biological effects (39). Hence, SNO had the therapeutic potential as a PTP1B inhibitor. However, it is unclear whether the CSNO effect was solely dependent on PTP1B inhibition. Other mechanisms may also be involved, and further investigation is needed. Meanwhile, whether there are other metabolites of SNO playing a role in myocardial protective effects still needs further exploration.

In summary, the present study found that endothelium-derived SNO promoted cardiac function in DCM mice. We showed the facilitatory effect of SNO on glucose uptake, GLUT4 membrane translocation and the insulin signaling pathway. SNO protected cells against high glucose-induced damage through inhibition of oxidative stress and excessive autophagy and improvement of mitochondrial dysfunction. Together, our study provided new insight into the molecular mechanism behind DCM, with the potential to be used as a therapeutic target in the future.

## Data availability statement

The original contributions presented in the study are included in the article/supplementary material. Further inquiries can be directed to the corresponding author.

## Ethics statement

The animal study was reviewed and approved by TongJi Hospital Application for Ethical Approval for Research Involving Animals.

## Author contributions

It was SL and LP who conceived and designed the study. The majority of the experiments were conducted and analyzed by LP, and the manuscript was written by LP. During experimentation and analysis of results, MZ, SH, WS, TJ, DP, MW, JG, LM provide guidance and advice. YJ, BH, QW assisted with the animal experiment. Reagents, equipment, and advice were provided by JL and LL. The manuscript was reviewed, edited, and feedback was provided by SL and LL. Final approval of the manuscript was obtained from all authors. All authors contributed to the article and approved the submitted version.

## Funding

This work was supported by the National Natural Science Foundation of China [grant numbers 81974032, 82070396].

## Conflict of interest

The authors declare that the research was conducted in the absence of any commercial or financial relationships that could be construed as a potential conflict of interest.

## Publisher’s note

All claims expressed in this article are solely those of the authors and do not necessarily represent those of their affiliated organizations, or those of the publisher, the editors and the reviewers. Any product that may be evaluated in this article, or claim that may be made by its manufacturer, is not guaranteed or endorsed by the publisher.
